# Food for Infants

**Published:** 1874-08

**Authors:** Alex S. Von Mansfelde

**Affiliations:** 211 South Hoyne Street; Chicago


ARTICLE VII.—*Food for Infants. A Review of the Review.* By ALEX. S. VON MANSFELDE, Chicago.

Personally we would refrain from a reply to the reviewer's article, since our "little knowledge" (we never claimed more) forbids us to curtail the space of a journal, the tendency of which is the dissemination of medical knowledge, either theoretical or practical; but for the desire to defend a theory, the practical demonstration of which is so highly worthy of the attention of the best of our profession, since it is followed by consequences not altogether indicative of "absolute nonsense."

We admit that our "surface reading," in the language of the reviewer, would forbid a rejoinder to his profound (?) criticism, but for one short-coming of our friend, the utter want of sight; and since we sat side by side with him in old Rush once upon a time, we are pained to observe this deformity, and, for old acquaintance sake, shall endeavor to operate, and hope the best from it for our friend.

He says, on page 286, May No., Chicago Med. Journal:

"The young infant relies upon milk for both food and drink; hence it is important that the proportion of fluid and solid should correspond with its wants. We add a *little water* (our own) to make good these proportions, and not, as our author observes, for the purpose of 'diluting casein.'"

We never said that water was added solely to dilute caseine, nor gave our opinion in regard to the matter, but simply quoted authorities, and the reviewer attests the same "*to make good the proportion*," and we denied, and do now most positively deny the correctness of such practice.

That milk is dilutable, or its components, of which caseine is one, we know by the practice of the Chicago dairy-men; and that the proportion of its solids to the fluid can be brought in harmony with the relation existing between the different components of human milk by the addition of a little water, 10–20 parts of water to a thousand, we surely could not deny; but ridicule the addition of 1000 parts of water to 885 already existing, in order to make up for a deficiency of 10 to 20 parts. *And such is the authorized practice!*

We are not aware of the beneficial action that such water does assert upon the precipitate in the stomach, other than a solemn protest of the cloudy caseine, left behind by the cowardly retreat of the water, leaving the poor organ to do the best it can with such *product of infinite dilution.* It must certainly be beyond the reviewer's right to claim propriety for such dilution, until he shows its favorable action upon the digestive process.

We claimed the opposite, and gave our reason. Authorities: Todd and Bowman. " Drinks, as water and various other liquids, fermented or not, are doubtless likewise in great part absorbed in the same way as liquefied albuminous food from the surface of the stomach. (Page 563) it is, its blood vessels (Page 562). If milk be introduced into the stomach, its caseine is first coagulated and afterwards apparently dissolved. The solidified caseine seems gradually to melt down, and becomes absorbed." (Page 560. Dalton's Physiology, p. 133.)

" There is never much fluid found in the stomach during digestion of any article of food, but gastric juice is constantly secreted and constantly absorbed, together with the food digested by it." (Dalton's Physiology, p. 133.)

" An observant patient of mine with emphysema, tells me that she finds it a good rule never to drink with her meals." (Chambers on Indigestion, p. 90.)

" Drinks are immediately absorbed, or otherwise disposed of." (Carpenter Phys., p. 114. William Beaumont's Experiments, p. 97.)

" But it has some degree of importance in demonstrating the fact that a degree of solidity (of food) is necessary for the operation of this agent (pepsin)." Dr. W. Beaumont's Experiments, p. 146. Author's edition, 1833.

Again he says, on page 287, Chicago Medical Journal:

" Just how sugar is assimilated we cannot tell, but this we know that it is *essential*," (his own).

We have no objection to a little sugar, if the reviewer pleases, but never observed its helping the infant, who was fed by milk to which sugar was added; which in such cases is seldom regulated by the natural requirements, but is given far above its normal quantity.

Does the reviewer assert, upon his own experience, that craving sugar is a *natural* appetite? Would like to have him prove that sugar does not interfere with digestion, or tend to intestinal irritation. What has he got to say against the assertion that children may have *polyphagia and pica?*

Will not oil take the sugar's place in the economy of the child, and may it not be *less harmful* than sugar? Authorities:

"It is therefore quite natural to find that *any excess of sugar taken ready made*, induces discomfort in dyspeptic patients. It is undigested, and the greater part undergoes *acetic fermentation* by the second or third hour after it is eaten. During its fermentation it also encourages fermentation in other articles of food, and by its presence oleaginous food is apt to be rendered indigestible also. Great discomfort will sometimes go on for a long time from this cause without being suspected, and cease by the simple expedient of *leaving off a constituent of diet so little necessary as sugar.*" (Chambers on Indigestion, p. 52.) (Emphasis our own.) "The much greater frequency of softening of the stomach and intestines in infancy and early childhood than in adult age, and the great amount and the wider extent of the alterations, have received considerable elucidation from the researches of Dr Elsaesser. He found that a much more rapid action upon the animal tissues, than that exerted by the gastric juice, was put forth by any substance capable of undergoing the *acetous fermentation combined with pepsin* (our own). Such substances are furnished by the milk, as well as by the various farinaceous and *saccharine matters* (our own) on which infants almost exclusively subsist. The tendency of these substances to undergo the acetous fermentation, is checked by the presence of healthy gastric juice, while, as we know by experience, it takes place very readily in infants who are dyspeptic, and to a very marked degree in many cases of infantile diarrhœa." (West. Diseases of Infancy and Childhood, p. 485.)

On the same page our reviewer says: "We have yet to learn that simple redness of the mucous surface, which always occurs during digestion, is an inflammatory indication, *as stated by the writer*" (our own). Now, what does the reader imagine we think of such language, when compared with our own statements: "The acidity and chemical changes leave a reddened condition of the intestinal membrane, an inflammatory indication, not even noticed when chloride of sodium is substituted for sugar."

What has the reviewer to say to Drs. Chambers, Elsaesser and West? Is the fermentation process so complained of by the former, caused by saccharine or oleaginous food? Is the softening of the stomach and intestines, which is found in so many children after death, as stated by the latter authorities, caused by normal afflux of blood consequent upon normal digestion? Or is not dilation of the capillaries, abnormal rapidity of the blood current, reddening of the surface, transmigration of the white-blood corpuscles; increased egress of the liquor sanguinis; stasis, caused by grouping of the red corpuscles—in one word, *inflammation*—the necessary precursor of softening, which may be and often is caused by fermentation of saccharine aliment? How can the reviewer charge us with confounding the *third step* in the series, denoting the inflammatory process, with *inflammation itself?* He certainly leaves room for doubt as to any work on pathological histology ever having reached his little village.

We may have our own estimate of the "*high calling*" in which we are engaged, but should like our next reviewer to disprove our *assertions*, and not define the relation we hold to our mental balance sheet.

In regard to the experiments we instituted, of which the reviewer speaks in such delicate terms on page 289—which language may be well compared with a *deliberate knocking down* and asking the prostrate antagonist's pardon "*with due courtesy*"—we simply draw the reader's attention to the following:

On page 288 he says: "In No. 3, according to his observation, precipitation failed to take place." On the same page: "In No. 3 we are utterly at a loss to understand how the gentleman could so fail in correct observation."

Now observe our own statement, on page 141: "In No. 3 we observe small granules of precipitated caseine, smaller and compacter than those in No. 1, but the fluid retained its milky appearance." Do we say anything even similar to his statements, or did we leave such impression upon the reader? We simply wished to convey the fact that the precipitate in No. 3 was neither deposited upon the bottom of the vial nor raised to the surface of the fluid, but was held in suspension in the same—and surely our language does not permit any other definition—in the form of a true caseine precipitate.

But what we did claim and assert to-day, is, that the addition of oil to milk does, either in the stomach by natural coagulation or by artificial precipitation with any acid, give form of a coagulum, which is far more spongy than that produced by the omission of oil.

Why does our reviewer say nothing about the physical appearance of his precipitate?

Why does he not try to disprove Elsaesser's assertion, to which our investigation forms simply the missing link?

That the reviewer has no objection to Lehman's *theory* (our own), we consider farcical, as his objection would not weigh a grain one way or the other; but the renowned teacher, we claim, has a right to see the authority by whom *his facts* are degraded to simple *theories*. We have given authorities in our article, and do this in our present note, and shall not answer any argument, after this, that is not based upon experiments, or is corroborated by authorities, *at least as good as our own*, for we aver our dislike to swallowing assertions whole.

Now, in regard to the addition of oil, our reviewer says, on page 284:

"We are decidedly of the opinion that *no addition* (his own) would be far preferable to that which he recommends but does not tell us he has tested practically."

On page 287 he says of sugar: "That as an aliment it can largely take the place of fat."

Now, all these would not deserve mentioning, for they are assertions and opinions of the individual, and manufactured for the occasion, but for the test of practicability he requires.

Please read what Chambers says on indigestion, page 63:

"The effects of cod-liver oil become less and less a marvel the more we know of physiology. The instinctive desire shown by all nations for an oleaginous diet, and their association of substances of this nature with proverbial ideas of happiness in all ages, show the value of a certain amount of it to man's comfort. The butter and honey of the prophet, used as a phrase of royal food, and the constant reference in the Bible to oil as a luxury (though it could have been no rarity in a land of oil-olive), these are sufficient to prove its estimation among the Hebrews. The Hindoo laborer, when he devours his gallon of rice for a meal, will spend all the pice he can get on the clarified butter of the country, and 'as good as gher!' is his expression of unqualified admiration. It was a mistake in Baron Liebig to state that oily foods are disgustful to natives of hot climates. All races of men require them and seek after them; and the taste of the Esquimaux, so often quoted, depends mainly on the abundant supply of the article which the sea places at his disposal, coupled with a scantiness of other provisions. Throughout mankind there is an instinctive appreciation of the importance of this aliment, independent of accidental differences of nationality or locality. It seems felt to be, as science shows that it really is, a necessary material for the renewal of the tissues, and the desire for it becomes synonymous with a desire for augmented life.

"An easily assimilated oil comes, in fact, into the short list of life-giving articles in the pharmacopœia, for it is itself the material by which life is manifested. Here, under its use, beneficial influences are exerted throughout the whole body; old wounds and sores heal up; the harsh, wrinkled skin regains the beauty of youth; debilitating discharges cease, at the same time that the normal secretions are more copious; the mucous membranes become clear and moist, and are no longer loaded with sticky epithelium; the pulse, too, becomes firmer and slower, that is to say, more powerful, for abnormal quickness here is always proof of a deficient vitality. Such are the effects, perfectly consistent with physiology, of supplying a deficiency of molecular base for interstitial growth."

What has the reviewer to say to this perfect hymn, sung to the praise of fat by one so capable?

On page 156, vol. xxvi, *Medical and Surgical Reporter*, we refer to the necessity of fat for the formation of all cell growth; for without this material, cell formation would not take place, at least such seems to be the conclusion arrived at by Rindfleisch and other histologists.

Dr. Burritt and myself, in *Medical and Surgical Reporter*, vols. xxiii and xxvi, have expressed our confidence in the efficacy of oil as a remedy for summer-complaint.

Dr. Fred. Schaller, of this city, has for years given almond emulsion with the most happy consequences, and *for the emulsified oil it contains*—for what else is of efficacy in this preparation?

He being a close observer, and having a large German practice, should surely be competent to witness the success of his remedy, and his praise is unqualified.

As to the efficacy of oil as an adjuvant to cows' milk, used for feeding infants, we refer the reviewer to the physical changes which it produces, and to its physiological effects, reminding him of the remarkable fact, that oil will act as a remedy in most disorders that are the cause and consequence of perverted digestion. Are not most hand-fed children sufferers from indigestion, and are not half of them victims of its deadly consequences?

What about the reviewer's rules?

No. 1 simply states what teachers and books, with very few exceptions, teach up to this day. And what does this signify? It simply asserts the loss of fifty infants out of a hundred by this practice, and proclaims the unsoundness of such teaching.

No. 2 asserts things which the reviewer cannot sustain—which are disproven by most eminent observers, as above cited, and may be shown to be incorrect by any one who is addicted to the use of much sugar, especially by candy-eaters.

No. 3 simply throws the reviewer's argument to the winds, for it gravely asserts everything we desire for the support of our position. We pity the reviewer for not making this statement the heading of his article; it would have saved him all the rest of it. It is: "If the milk be properly selected"—in the meaning of the reviewer, the last of the milking—"it is not necessary to add oil to it, for it contains already more fat than human milk." How wise!

*My dear Doctor, this is the very thing we want!*

*Please send us, by all means, the last milking of all your cows, and then, farewell to "detestably adulterated cod-liver oil and sweet almond oil"!*

Rule No. 4 has nothing to do with our article, for we would not breathe a word against it, nor did we.

Finally, we express our thanks to the reviewer for having noted our weak effort to further the cause of poor, suffering baby. And if we most humbly bow to Him who in the deep holds hidden many a ray of the sun of truth, we nevertheless strive to pluck one by one from their secret recesses to enlighten the world of science, in twilight arrayed, and hope that a glimmer of it will not fail to reach Low Point.

And should the warming tendency of the advance of knowledge melt away the crust that enshrines so many hearts, endeavoring, from their limited boundaries, to pluck the credit from honest endeavor, the writer will be amply rewarded; and the CHICAGO MEDICAL JOURNAL, for undertaking the laborious task of mediator, may be assured of the thanks of many who in their time have drunk only too much of brotherly love enshrined in calcified hearts.

211 SOUTH HOYNE STREET.

